# Associations between household chaos and appetitive traits in preschoolers and preadolescents

**DOI:** 10.3389/fpubh.2026.1800392

**Published:** 2026-09-21

**Authors:** Zhuoya Zhang, Delaina Carlson, Caroline Borowy, Mingliang Ge, Diane Gilbert-Diamond, Jennifer A. Emond

**Affiliations:** 1 Department of Epidemiology, Geisel School of Medicine, Dartmouth College, Hanover, NH, United States; 2 Department of Human Development and Family Sciences, University of Connecticut, Mansfield, CT, United States; 3 Department of Pediatrics, Geisel School of Medicine, Dartmouth College, Hanover, NH, United States; 4 Department of Medicine, Geisel School of Medicine, Dartmouth College, Hanover, NH, United States; 5 Department of Biomedical Data Science, Geisel School of Medicine, Dartmouth College, Hanover, NH, United States

**Keywords:** middle childhood, appetitive trait, childhood obesity, eating behavior, emotional eating, food responsiveness, household chaos, preschool

## Abstract

**Introduction:**

Prior research suggests that household chaos may relate to greater childhood obesity risk, though mechanisms are unclear. This study aimed to evaluate the cross-sectional associations between household chaos and appetitive traits in 92 preschoolers (Study A) and 184 preadolescents (Study B).

**Methods:**

Parents completed the Confusion, Hubbub, and Order Scale and the Child Eating Behavior Questionnaire. Eating in the absence of hunger (EAH) was assessed in the lab. Data were collected at baseline and at either 6-month (Study A) or 1-year (Study B) follow-up. Associations between household chaos and appetitive traits were examined cross-sectionally using linear mixed-effects models that accounted for repeated observations within children and adjusted for child age, sex, and annual household income. Sensitivity analyses further adjusted for nighttime sleep duration and parenting style in both studies, and the number of children in the household in Study A only.

**Results:**

In both cohorts, chaos was positively associated with emotional overeating (preschoolers: standardized beta coefficient *β*_*s*_= 0.18, 95% CI: 0.005, 0.35, *p* = 0.04; preadolescents: *β*_*s*_= 0.19, 95% CI: 0.07, 0.31, *p* = 0.003). In preadolescents, more household chaos was associated with higher food responsiveness (*β*_*s*_ = 0.21, 95% CI: 0.09, 0.32, *p* < 0.001) and lower satiety responsiveness (SR, *β*_*s*_ = −0.14, 95% CI: −0.25, −0.02, *p* = 0.03). No associations were found for enjoyment of food, emotional undereating, or EAH.

**Conclusions:**

Household chaos was associated with several appetitive traits linked to obesity risk, particularly emotional overeating across both age groups and greater food responsiveness and lower satiety responsiveness in preadolescents. These findings suggest that chaotic home environments may shape children's eating behaviors and represent a potential modifiable target for obesity prevention.

## 1 Introduction

Appetitive traits, defined as predispositions to approach or avoid food (1), can contribute to childhood obesity. A meta-analysis of epidemiologic studies indicates that food approach appetitive traits, including food responsiveness (FR, i.e., responsivity to food cues, *r* = 0.22) and emotional overeating (EOE, *r* = 0.15), are associated with higher weight status in children aged 1 month to 13 years (2). Eating in the absence of hunger (EAH) is conceptualized as consuming palatable foods beyond satiation (3). EAH can be operationalized as lab-observed intake of palatable foods following a satiating meal (4). Longitudinal and intervention studies suggest that EAH is related to feeding environment (5) and can be modified with cue reactivity training (6). A systematic review of epidemiological studies indicates that EAH is associated with higher weight status among children aged 12 years or younger (4). In contrast, food avoidance appetitive traits, such as satiety responsiveness (SR, i.e., the tendency to stop eating when feeling full), are associated with lower weight status (*r* = −0.21) in children aged 1 month to 13 years (2). Beyond adiposity, higher FR and EOE and lower SR have been associated with less favorable cardiometabolic profiles in adolescents aged 12 years (7). Despite these health implications, little is known about the modifiable environmental factors related to children's appetitive traits.

The Family Ecological Model posits that the family context shapes the development of child health behaviors (8). A key dimension of this context is household chaos, which refers to the level of instability (i.e., hurriedness and lack of family routines) and disorganization (i.e., high background noise, crowding, and clutter) within the household (9). A structured home can offer children consistent routines around daily activities, such as mealtime and sleep (10–12). In contrast, household chaos can make it difficult for children to engage in regular, healthful behaviors (13). Further, chaos has been associated with lower executive functioning (14), lower self-regulation (15), and higher weight status in young children (16, 17), suggesting that chaos may affect mechanisms related to impulsive eating and overeating among children.

While chaos has been linked to a range of adverse child health outcomes (18), its relationship with children's appetitive traits is less clear. Two studies have examined this question among young children (19, 20). Ju et al. (20) found that chaos was related to more emotional overeating in children aged 18–24 months (*N* = 371). Lumeng et al. observed in children aged 3–4 years (*N* = 331) that chaos was associated with reduced satiety responsiveness in females and increased emotional overeating in males, suggesting that sex may modify the association between chaos and appetitive traits (19). However, these findings have not been confirmed with lab-observed measures of hedonic eating. Further, the relationship between household chaos and appetitive traits in middle childhood remains unexplored. One potential pathway is through sleep, as chaos has been associated with reduced sleep duration and quality in children (21, 22), and insufficient sleep has been linked to greater food responsiveness (23) and lower satiety responsiveness (24). Understanding the impact of household chaos on children's appetitive traits can inform effective childhood obesity interventions that target modifiable aspects of the household environment.

This study aims to evaluate the associations between household chaos and appetitive traits (parent-reported and lab-observed) in independent samples of preschool-aged children and preadolescents. We hypothesized that in both groups, (a) household chaos would be positively associated with food responsiveness, emotional overeating, enjoyment of food, and EAH, and (b) household chaos would be negatively associated with satiety responsiveness and emotional undereating.

## 2 Materials and methods

### 2.1 Study samples

This study is a secondary analysis of data collected from two independent cohorts. Data include (1) parent responses to questionnaires and (2) lab-observed eating in the absence of hunger. Study A collected data at baseline (*N* = 92) and a 6-month follow-up (*N* = 80). Study B collected data at baseline (*N* = 184) and a 1-year follow-up (*N* = 95). Participants who completed the follow-up visits had demographics similar to those who completed the baseline visit (*p* > 0.05; Supplementary Table S1).

Study A enrolled children aged 3–5 years and a parent (including guardians) via flyers and email listservs from New Hampshire and Vermont from July 2018 through October 2022. Participants were informed of the study's primary objective in the consent form: to evaluate the impact of media use on young children's sleep and eating behaviors. Children were eligible if they could comprehend English; had no significant developmental or language delay, sleep disorders, food allergies, or dietary restrictions, appetite- or attention-altering medications or disorders; had no relatives in this study; had no plan to relocate in 6 months; and lived with the enrolled parent for more than half the time. Participants were compensated with $60 in e-gift cards per visit (two visits in total: baseline and month-6 follow-up). Parents provided written consent, and children provided verbal assent at baseline.

Study B enrolled children aged 9–12 years and a parent (including guardians) from the same geographical areas as Study A from April 2018 through April 2023. The original study aimed to examine the association between food-cue-related neural reward reactivity and EAH behavior and potential effect modification by obesity-related genotypes. Parents were informed of the study's primary aim in the consent form. Children were eligible if they could comprehend English; had no relevant food allergies or dietary restrictions; did not take appetite- or attention-altering medications and did not have appetite- or attention-altering conditions, psychiatric disorders, or neurological disorders; had no relatives in the study; and were willing to participate in all study visits. Because this study involved magnetic resonance imaging, children were excluded if they had metal in or on their bodies or if they had claustrophobia. Participants were compensated $55 in e-gift cards for completing their baseline visit and $65 for completing their one-year follow-up visit. Parents provided informed written consent, and children provided informed written assent at baseline. Dartmouth College's Committee for the Protection of Human Subjects approved the study protocols (Study A: CPHS31097; Study B: CPHS30723).

### 2.2 Exposure variable: household chaos

In both studies, parents completed the Confusion, Hubbub, and Order Scale (CHAOS), a validated unidimensional questionnaire at baseline and follow-up visits (25). Parents rated their own household on 15 statements describing characteristics of a chaotic household environment, e.g., “No matter how hard we try, we always seem to be running late” and “Our home is a good place to relax” (reverse scored). Responses range from 1 (very much like your own home) to 4 (not at all like your own home). Total scores were the sum of item scores, ranging from 15 to 60. Higher scores indicate a more chaotic household. The scale showed acceptable internal consistency in the original study (Cronbach's α = 0.79) and in our samples (Study A: α = 0.85; Study B: α = 0.87) (25) (Supplementary Table S2). The scale showed high one-year test-retest reliability (*r* = 0.74) and convergent validity against observed measures of household organization in the original study (25). While unrelated to socioeconomic status (SES) in the original study (25), the scale correlated with the number of children in the household (Supplementary Table S3), annual household income (Supplementary Table S4), and homeownership status in our samples and in another study among preschoolers (12).

### 2.3 Outcome variables

#### 2.3.1 Parent-reported child appetitive traits

Parents completed the Child Eating Behavior Questionnaire (CEBQ) to assess children's appetitive traits at baseline and follow-up visits (26). Our analysis considered three subscales on food approach traits: food responsiveness (FR, five items, e.g., “If allowed to, my child would eat too much.”), emotional overeating (EOE, four items, e.g., “My child eats more when worried.”), and enjoyment of food (EF, four items, e.g., “My child loves food.”). Two food avoidance traits were also assessed: satiety responsiveness (SR, five items, e.g., “My child gets full before his/her meal is finished.”) and emotional undereating (EUE, four items, e.g., “My child eats less when angry.”). Parents rated each item on a five-point Likert scale from 1 (never) to 5 (always). Subscale scores were calculated as the average of item responses (range: 1–5). Higher scores indicate a greater perceived presence of the appetitive traits by parents. Subscales showed acceptable to high internal consistency in our samples (Study A: α range: 0.69–0.91; Study B: 0.77–0.83) (Supplementary Table S2).

#### 2.3.2 Lab-observed eating in the absence of hunger

In Study A (baseline and 6-month follow-up), children completed the eating in the absence of hunger experiment in our lab, following an EAH protocol previously established by our group (27). Children were first served with a preload snack (50 g peeled banana, 58 g sliced cheese, 28 g crackers, and water) and were instructed to eat until satiated. They then rated their fullness on a three-point visual analog scale (1 = hungry, 2 = half-hungry/half-full, 3 = full). Next, children were presented with two snacks in open bowls, one savory and one sweet (60 ± 5 g Goldfish crackers and 60 ± 5 g mini graham crackers), and were instructed to eat *ad libitum* while watching a standardized 17-min TV program featuring food commercials.

In Study B (baseline and 1-year follow-up), children first received a preload meal (macaroni and cheese, apple sauce, corn, milk, and water), served in a fixed caloric ratio, with the total amount scaled to approximately 25% of each child's estimated daily energy requirement (28). They were instructed to eat until satiated and offered more if still hungry. They then reported their fullness on a validated visual analog scale (Freddy's Fullness Scale) (29). Next, they were presented with snacks in open bowls spanning savory, sweet, and healthy options (240 ± 1 g gummy bears, 76 ± 0.5 g Goldfish crackers, 235 ± 1 g grapes, and water) and instructed to eat *ad libitum* while watching a standardized 35-min TV show embedded with food commercials (28).

In both studies, trained research staff weighed all snacks before and after the EAH phase. EAH was operationalized as the total caloric intake during the experiment, computed by multiplying each snack's known energy content per gram by the amount consumed.

### 2.4 Covariates

#### 2.4.1 Demographics

In both studies, parents reported their children's dates of birth, sex, race, and ethnicity on the baseline questionnaire. Parents reported annual household income at baseline. In Study A, parents also reported the number of children living in the household at baseline.

#### 2.4.2 Parenting style

In Study A, parents self-reported parenting style with the Parenting Style and Dimensions Questionnaire short form (PSDQ) at baseline (30). Study A considered permissive parenting (five items, e.g., “I find it difficult to discipline my child,” α = 0.68) and authoritative parenting subscale (15 items, e.g., “I give my child reasons why rules should be obeyed,” α = 0.85). Both subscales range from 1 to 5, with higher scores indicating greater permissive or authoritative parenting styles, respectively.

In Study B, children assessed their parents' parenting style with the Authoritative Parenting Index (API) at baseline (31). The API has two subscales. Demandingness was assessed with four items, e.g., “She/he has rules that I must follow,” with subscale scores ranging from 4 to 16 (α = 0.52). Responsiveness was evaluated with five items, e.g., “She/he listens to what I have to say,” with subscale scores ranging from 5 to 20 (α = 0.75). Higher scores reflect more responsive or demanding parenting.

#### 2.4.3 Nighttime sleep duration

Actigraphy was used to collect children's sleep patterns in the home environment at baseline and follow-up visits in both studies (24). Raw accelerometry data were processed separately for each cohort due to the age difference. Study A participants wore ActiGraph wGT3X-BT devices (Pensacola, FL) on their non-dominant ankles 24 h per day for at least five consecutive days. Parents reported their child's bedtime and wake time on a sleep log. We used the Sitnik algorithm to detect wake time if it was missing from the sleep log (32). Parents also recorded any non-wear periods, which were excluded from the analysis. The nighttime sleep period was identified as the period from parent-reported bedtime to wake time the next morning. Within that window, each minute was classified as sleep or wake with the Sadeh algorithm (33, 34). Study B participants wore Actigraph GT9X Link devices on their non-dominant wrists for at least 5 days. Raw accelerometry data were processed into 60-s epochs on the ActiLife 6 software (35). Each minute was marked as sleep or wake with the Cole-Kripke algorithm (36). Sleep periods (from bedtime to wake time the next morning) were identified using the Tudor-Locke algorithm (37). Non-wear periods were detected with the Troiano algorithm and were excluded from the analysis (38).

Nighttime sleep duration was computed by summing the minutes marked as asleep for each overnight sleep period. In both studies, participants with at least 3 nights of valid data were included in the analysis (Study A: baseline *N* = 73, month 6 *N* = 50; Study B: baseline *N* = 154, one-year *N* = 79). The average nighttime sleep duration was computed as the mean across all valid nights.

#### 2.4.4 Anthropometry

Trained research staff measured participant weight to the nearest 0.1 kg and height to the nearest 0.1 cm three times using a Seca 703 Medical Scale and Seca 264 Stadiometer (Hamburg, Germany) during in-person visits. Study A collected anthropometry at baseline only, because in-person visits were paused during the COVID-19 pandemic (*N* = 52). Study B collected anthropometry at baseline (*N* = 183) and 1-year follow-up (*N* = 94). Age- and sex-standardized body mass index *z*-scores (BMI-z) were computed using the 2000 CDC growth charts (39).

### 2.5 Statistical analysis

First, we computed descriptive statistics of all variables to summarize participants' baseline characteristics. Next, we used Pearson's Chi-squared tests and Welch's two-sample *t*-tests to compare the distribution of sociodemographic characteristics between participants in the baseline samples and those who completed the follow-up visits. Additionally, we conducted bivariate analyses of the association between household chaos and participant baseline demographics using the Spearman correlation test, the Wilcoxon rank-sum test, or the Jonckheere–Terpstra test for trend, as appropriate. Lastly, we fit linear mixed-effects models with random intercepts to estimate the cross-sectional association between household chaos and each appetitive trait, accounting for repeated measures within each child. Child age, sex, and annual household income were included in all models, as child appetitive traits may vary by age and sex (40, 41) and household income was related to chaos in our sample (Supplementary Table S4) and in prior work (42).

Sensitivity analyses further adjusted for parenting style (43) and child nighttime sleep duration (24) (both studies) and number of children in the household (44) (Study A only). We created a directed acyclic graph to identify potential confounders and mediators (Supplementary Figure S1). Bivariate analyses were conducted to examine the correlations across child nighttime sleep duration, appetitive traits, adiposity, and household chaos. Further, we tested potential effect modification by child sex by including an interaction term between chaos and child sex in the models. Additionally, the association between household chaos and child BMI-z was examined using linear regression or linear mixed-effects models, as appropriate. The intra-class correlation coefficient (ICC; two-way random-effects model) was computed for household chaos and appetitive traits to assess the stability of these constructs over time. Based on past research, an ICC between 0.50 and 0.75 was considered moderate, 0.75 and 0.90 reasonable, and 0.90 or higher excellent reliability (45). All statistical analyses were conducted with R version 4.3.2 (R Core Team, 2023). A *p*-value < 0.05 was considered statistically significant.

## 3 Results

### 3.1 Participant characteristics

In Study A, participants were, on average, 4.2 years old (SD = 1.0), with 44% female at baseline (Table 1). In Study B, the average participant baseline age was 10.9 (SD = 1.2), with 42% female. In both samples, most participants were White and non-Hispanic (Study A: 88%; Study B: 90%). In both samples, most children were from households with annual incomes above $65,000 (Study A: 72%; Study B: 87%). Twenty-three percent of preschoolers and 31% of preadolescents were with overweight or obesity.

**Table 1:** Participant baseline characteristics.

Characteristic	Study A: preschoolers (*N* = 92)	Study B: preadolescents (*N* = 184)
Child age (years)	4.2 ± 1.0	10.9 ± 1.2
Female	40 (44%)	77 (42%)
White non-Hispanic	81 (88%)	166 (90%)
With overweight or obesity^a^	12 (23%)	57 (31%)
Annual household income ($)^b^		
< 65,000	26 (28%)	24 (13%)
65,000–145,000	47 (51%)	99 (54%)
145,000–225,000	13 (14%)	43 (23%)
>225,000	6 (7%)	18 (10%)
Household chaos (CHAOS)^c^	28.2 ± 6.6	28.5 ± 7.1
Average sleep duration (hours)^d^	9.0 ± 0.9	7.0 ± 1.0

Mean ± SD or *N* (%) are presented in the table.

^a^Weight and height were collected at baseline for 52 children in Study A and 183 children in Study B.

^b^Household income was missing for four Study A participants and was imputed with the sample mode.

^c^Household chaos was assessed with the Confusion, Hubbub, and Order Scale (CHAOS, range: 15–60).

^d^Assessed with accelerometry (Study A: *n* = 73; Study B: *n* = 154).

The average score on the CHAOS scale was 28.2 (SD = 6.6) in Study A and 28.5 (SD = 7.1) in Study B. In Study A, families with more children living in the household scored higher on the CHAOS scale (*p* < 0.001; Supplementary Table S3). In Study B, lower household income was associated with greater household chaos (*p* = 0.003; Supplementary Table S4).

The average accelerometer-assessed nighttime sleep duration was 9.0 h (SD = 0.9) in Study A participants and 7.0 (SD = 1.0) in Study B participants (Table 1). Sleep duration was not correlated with household chaos in either cohort (Supplementary Figures S2, S3). Sleep duration was positively correlated with satiety responsiveness (*r* = 0.20, *p* < 0.05) and emotional undereating (*r* = 0.17, *p* < 0.05) in preadolescents (Supplementary Figure S3). No correlations between sleep duration and appetitive traits were found in preschoolers (Supplementary Figure S2).

### 3.2 Stability of household chaos and appetitive traits

Household chaos showed high stability in Study A (6-month ICC = 0.93) and Study B (1-year ICC = 0.85) (Supplementary Table S5). Additionally, parent-reported appetitive traits showed moderate to high stability, with 6-month ICCs ranging from 0.56 (EUE) to 0.77 (FR) in preschoolers and one-year ICCs ranging from 0.59 (EOE and EUE) to 0.74 (FR) in preadolescents (Supplementary Table S5).

### 3.3 Associations between household chaos and appetitive traits

Household chaos was positively associated with emotional overeating in both preschoolers (standardized beta coefficient *β*_*s*_ = 0.18, 95% CI: 0.005, 0.35, *p* = 0.04) and preadolescents (*β*_*s*_ = 0.19, 95% CI: 0.07, 0.31, *p* = 0.003; Figure 1, Table 2). In the sensitivity analysis in preschoolers, this association was attenuated toward the null after further adjusting for the number of children in the household (Supplementary Table S6), whereas in both cohorts it remained similar after further adjustment for parenting style and nighttime sleep duration (Supplementary Tables S6, S7).

**Figure 1:**
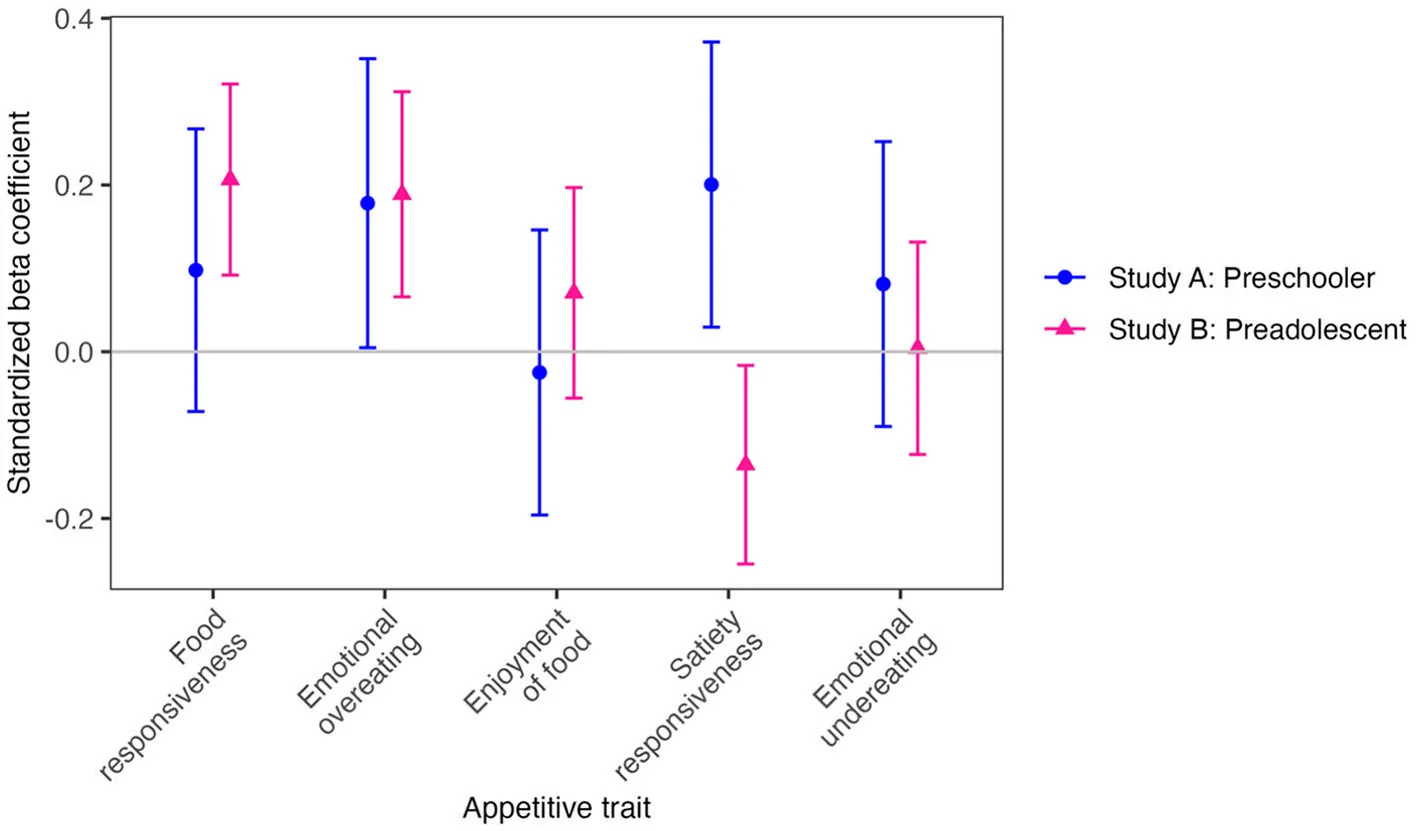
Associations between household chaos and appetitive traits in preschoolers and preadolescents. Standardized beta coefficients were from linear mixed-effects models with household chaos as the independent variable and each appetitive trait as the dependent variable. Models were adjusted for child age, sex, and annual household income. Error bars present the 95% confidence intervals of the beta coefficients.

**Table 2:** Associations between household chaos and appetitive traits in preschoolers and preadolescents.

Outcome variable	Study A: preschoolers (*N* = 92)		Study B: preadolescents (*N* = 184)	
	*β*_*s*_ (95% CI)	*p*	*β*_*s*_ (95% CI)	*p*
Food responsiveness	0.10 (−0.07, 0.27)	0.26	0.21 (0.09, 0.32)	< 0.001
Emotional overeating	0.18 (0.005, 0.35)	0.04	0.19 (0.07, 0.31)	0.003
Enjoyment of food	−0.02 (−0.20, 0.15)	0.77	0.07 (−0.06, 0.20)	0.27
Satiety responsiveness	0.20 (0.03, 0.37)	0.02	−0.14 (−0.25, −0.02)	0.03
Emotional undereating	0.08 (−0.09, 0.25)	0.35	0.00 (−0.12, 0.13)	0.95
EAH^a^	−0.03 (−0.25, 0.18)	0.76	0.06 (−0.06, 0.18)	0.30

Table presents standardized beta coefficients (*β*_*s*_) from linear mixed-effects models, adjusted for child age, sex, and annual household income.

^a^EAH, caloric intake during the eating in the absence of hunger experiment. EAH was only collected in 70 participants in Study A, because in-person visits were paused during the COVID-19 pandemic. *N* = 92 for all other outcomes in Study A.

Household chaos was also positively associated with food responsiveness in preadolescents (*β*_*s*_= 0.21, 95% CI: 0.09, 0.32, *p* < 0.001; Figure 1, Table 2). This association remained similar after further adjustment for parenting style and sleep duration (Supplementary Table S7). No association between chaos and food responsiveness was observed in preschoolers (Table 2).

The association between household chaos and satiety responsiveness differed by age group (Figure 1, Table 2). In preadolescents, greater chaos was associated with lower satiety responsiveness (*β*_*s*_ = −0.14, 95% CI: −0.25, −0.02, *p* = 0.03), and this inverse association remained similar after adjustment for parenting style and sleep duration (Supplementary Table S7). In preschoolers, by contrast, greater chaos was associated with higher satiety responsiveness (*β*_*s*_ = 0.20, 95% CI: 0.03, 0.37, *p* = 0.02), yet this positive association became non-significant after adjustment for sleep duration.

Household chaos was not associated with enjoyment of food, emotional undereating, eating in the absence of hunger (Table 2), or BMI-z (Supplementary Table S8) in either cohort (all *p* > 0.05), and these null findings were unchanged across all sensitivity analyses (Supplementary Tables S6, S7). Lastly, child sex did not modify the association between household chaos and any appetitive trait in either cohort (all interaction *p* > 0.10; Supplementary Table S9).

## 4 Discussion

This study examined the cross-sectional associations between household chaos and appetitive traits in two cohorts of children in early and middle childhood, primarily from higher-SES families. Higher household chaos was associated with greater emotional overeating in both preschoolers and preadolescents. Additionally, chaos was associated with increased food responsiveness and decreased satiety responsiveness in preadolescents. Longitudinal and experimental studies are needed to clarify temporality and underlying mechanisms.

Household chaos was positively related to emotional overeating in both preschoolers and preadolescents. This is consistent with prior research. Ju and colleagues reported a positive chaos-EOE association in children aged 18–24 months (*β*_*s*_= 0.12, *p* = 0.05) (20), and Lumeng and colleagues observed a similar association in children aged 3–4 years (*r* = 0.20, *p* < 0.05) (19). Increased stress and reduced executive functioning may explain the chaos-EOE association. Higher household chaos has been shown to increase child stress and negative affect (46, 47), which can in turn impair the development of self-regulation and executive functioning (48). Further, Hong and colleagues found that greater chaos was associated with lower emotion regulation in children aged 6–9 years (49). Emotional overeating may serve as a maladaptive coping mechanism for children to regulate negative affect (e.g., frustration, fear, anger) arising from household chaos (50–52). Mediation analysis should investigate the pathways linking household chaos to child emotional overeating.

Notably, further adjusting for family size attenuated the association between chaos and emotional overeating toward the null in preschoolers (Supplementary Table S6). The number of children living in the household was positively correlated with household chaos (Spearman's *r* = 0.51, *p* < 0.0001) and EOE (*r* = 0.28, *p* = 0.007) in Study A. We adjusted for this variable in the sensitivity analysis as a confounder, since it is correlated with both the exposure and the outcome variables and prior work has considered family size a correlate (53) rather than a component of the household chaos construct (25). However, we could not replicate this analysis in Study B, as family size was not assessed. Further longitudinal studies are needed to examine whether family size would confound the association between chaos and child appetitive traits.

Among preadolescents, greater household chaos was associated with increased food responsiveness and reduced satiety responsiveness (Table 2, Figure 1). These findings are consistent with prior research by Lumeng and colleagues (19), who reported a similar positive association between household chaos and FR (*r* = 0.21, *p* < 0.001) among preschool-aged children from lower socioeconomic backgrounds. Several mechanisms may explain this association. First, household chaos may increase physiological arousal and environmental distractions for children (54), potentially attenuating their sensitivity to internal satiety signals. Second, the lack of household routines may disrupt children's circadian rhythms and appetite regulation (55), increasing their reactivity to external food cues (56, 57). These mechanistic hypotheses need to be investigated in experimental studies.

Contrary to our hypothesis, we observed a positive association between household chaos and satiety responsiveness (standardized *β*_*s*_ = 0.20, 95% CI: 0.03, 0.37; *p* = 0.02) in preschoolers. This finding was unexpected and was in the opposite direction from the inverse association we observed in Study B. To our knowledge, a positive chaos-SR relationship has not been reported previously. Several explanations are possible. First, because household chaos and satiety responsiveness were both reported by the same parent, the association may partly reflect common-method bias. Parents in busier, more chaotic households may perceive and report more frequent meal-ending or fullness behaviors in their children. Second, unmeasured factors, such as parental feeding practices (e.g., pressure to eat), were not assessed here and could contribute to unmeasured confounding. Lastly, when sleep was further adjusted in the model, the association was attenuated toward the null (*β*_*s*_= 0.13, 95% CI: −0.08, 0.33; *p* = 0.22) (Supplementary Table S7). However, a *post hoc* analysis revealed that this attenuation was primarily due to data restrictions (smaller sample with available sleep data; *N* = 73) rather than to sleep adjustment itself. The chaos-SR relationship remained non-significant in this restricted sample even without sleep adjustment (*β*_*s*_ = 0.16, 95% CI: −0.07, 0.39; *p* = 0.16). Given these considerations, the positive chaos-SR association in preschoolers should be interpreted with caution and requires replication.

### 4.1 Strengths and limitations

This study has strengths and limitations. A key strength is that our study samples included children at two distinct developmental stages (early and middle childhood), extending the literature on the associations between household chaos and appetitive traits in older children (18). Repeated measurements allowed us to assess the stability of household chaos and child appetitive traits over time and improve statistical power. Several limitations should also be considered. First, household chaos and appetitive traits were assessed using questionnaires from the same parent, which may increase the risk of response bias (58). Future studies should incorporate multiple informants. Additionally, the API demandingness subscale showed low internal consistency (Cronbach's α = 0.52) in the preadolescent sample. In a sensitivity analysis that adjusted only for the API responsiveness subscale, the results remained similar (Supplementary Table S7). The CHAOS scale focuses on assessing the physical and organizational features of the home environment and does not evaluate the affective quality of family relationships, such as family cohesion and conflicts in parent-child interactions. Future studies could examine how these factors relate to children's appetitive traits. Second, while both Study A and Study B provided savory and sweet snacks to children in the EAH experiment, the food items differed between studies, potentially affecting palatability. Thus, the absolute EAH energy intake may not be directly comparable between the two cohorts. Third, incomplete sleep data in Study A and the study's cross-sectional design limited our ability to examine sleep as a potential mediator of the association between household chaos and children's appetitive traits. Formal mediation analyses using longitudinal data are needed to test whether insufficient or poor-quality sleep links household chaos to child appetitive traits. Further, children in both samples were primarily White, non-Hispanic, of higher SES, and without overweight or obesity, which may limit generalizability of our findings. Lastly, the cross-sectional design of both Study A and Study B precludes causal inference, and we cannot rule out reverse causation (i.e., children's appetitive traits contributing to household chaos rather than the reverse). Longitudinal and experimental studies with larger, more racially and socioeconomically diverse samples are needed to confirm these findings.

## 5 Conclusion

In both preschoolers and preadolescents, greater household chaos was associated with higher emotional overeating. In preadolescents, greater chaos was also associated with higher food responsiveness and lower satiety responsiveness. These findings suggest that household chaos may be a modifiable target for childhood obesity prevention and intervention efforts. Longitudinal studies are needed to establish causality, and intervention studies should examine whether reducing household chaos would improve children's eating behaviors and weight outcomes.

## Data Availability

The raw data supporting the conclusions of this article will be made available by the authors, without undue reservation.

## References

[1] Carnell S Benson L Pryor K Driggin E. Appetitive traits from infancy to adolescence: using behavioral and neural measures to investigate obesity risk. Physiol Behav. (2013) 121:79–88. doi: 10.1016/j.physbeh.2013.02.015 23458627 PMC3725261

[2] Kininmonth A Smith A Carnell S Steinsbekk S Fildes A Llewellyn C. The association between childhood adiposity and appetite assessed using the Child Eating Behavior Questionnaire and Baby Eating Behavior Questionnaire: a systematic review and meta-analysis. Obes Rev Off J Int Assoc Study Obes. (2021) 22:e13169. doi: 10.1111/obr.13169 33554425

[3] Fisher JO Birch LL. Restricting access to foods and children's eating. Appetite. (1999) 32:405–19. doi: 10.1006/appe.1999.0231 10336797

[4] Lansigan RK Emond JA Gilbert-Diamond D. Understanding eating in the absence of hunger among young children: a systematic review of existing studies. Appetite. (2015) 85:36–47. doi: 10.1016/j.appet.2014.10.032 25450900 PMC4277716

[5] Birch LL Fisher JO Davison KK. Learning to overeat: maternal use of restrictive feeding practices promotes girls' eating in the absence of hunger. Am J Clin Nutr. (2003) 78:215–20. doi: 10.1093/ajcn/78.2.215 12885700 PMC2530927

[6] Boutelle KN Zucker NL Peterson CB Rydell SA Cafri G Harnack L. Two novel treatments to reduce overeating in overweight children: a randomized controlled trial. J Consult Clin Psychol. (2011) 79:759–71. doi: 10.1037/a0025713 22122291 PMC3989524

[7] Zhang Z Li N Buckley JP Cecil KM Chen A Eaton CB . Associations between eating behaviours and cardiometabolic risk among adolescents in the health outcomes and measures of the environment study. Pediatr Obes. (2023) 18:12979. doi: 10.1111/ijpo.12979 36094787 PMC10392767

[8] Davison KK Jurkowski JM Lawson HA. Reframing family-centred obesity prevention using the Family Ecological Model. Public Health Nutr. (2013) 16:1861–9. doi: 10.1017/S1368980012004533 23089267 PMC5500251

[9] Evans GW Wachs TD editors. Chaos and Its Influence on Children's Development: An Ecological Perspective [Internet]. Washington, DC: American Psychological Association (2010). Available online at: https://www.jstor.org/stable/j.ctv1chs0mk (cited November 18, 2023).

[10] Emond JA. Household chaos: a risk factor for adverse child outcomes gains attention in public health. BMC Public Health. (2020) 20:596. doi: 10.1186/s12889-020-08680-y 32357857 PMC7193378

[11] Larsen KL Jordan SS. Organized chaos: daily routines link household chaos and child behavior problems. J Child Fam Stud. (2020) 29:1094–107. doi: 10.1007/s10826-019-01645-9

[12] Emond JA Tantum LK Gilbert-Diamond D Kim SJ Lansigan RK Neelon SB. Household chaos and screen media use among preschool-aged children: a cross-sectional study. BMC Public Health. (2018) 18:1210. doi: 10.1186/s12889-018-6113-2 30373557 PMC6206857

[13] Bates CR Buscemi J Nicholson LM Cory M Jagpal A Bohnert AM. Links between the organization of the family home environment and child obesity: a systematic review. Obes Rev. (2018) 19:716–27. doi: 10.1111/obr.12662 29520946

[14] Andrews K Atkinson L Harris M Gonzalez A. Examining the effects of household chaos on child executive functions: a meta-analysis. Psychol Bull. (2021) 147:16–32. doi: 10.1037/bul0000311 33151702

[15] Zhu H Shu L Wang X Xu Z. Household chaos and preschool migrant children's self-regulation: the mediating role of parent–child conflict and the moderating role of mindful parenting. Front Psychol. (2025) 16:1416040. doi: 10.3389/fpsyg.2025.1416040 40497109 PMC12150784

[16] Riley HO Lo SL Rosenblum K Sturza J Kaciroti N Lumeng JC . Sex differences in the association between household chaos and body mass index z-score in low-income toddlers. Child Obes. (2020) 16:265–73. doi: 10.1089/chi.2019.0186 32155340 PMC7262641

[17] Khatiwada A Shoaibi A Neelon B Emond JA Benjamin-Neelon SE. Household chaos during infancy and infant weight status at 12 months. Pediatr Obes. (2018) 13:607–13. doi: 10.1111/ijpo.12395 30019385 PMC6300983

[18] Marsh S Dobson R Maddison R. The relationship between household chaos and child, parent, and family outcomes: a systematic scoping review. BMC Public Health. (2020) 20:513. doi: 10.1186/s12889-020-08587-8 32316937 PMC7175577

[19] Lumeng JC Miller A Peterson KE Kaciroti N Sturza J Rosenblum K . Diurnal cortisol pattern, eating behaviors and overweight in low-income preschool-aged children. Appetite. (2014) 73:65–72. doi: 10.1016/j.appet.2013.10.016 24177439 PMC3895450

[20] Ju S Iwinski S Fiese BH McBride BA Bost KK. Influences of child temperament and household chaos on preschoolers' emotional eating. Child Obes. (2022) 18:523–32. doi: 10.1089/chi.2021.0237 35231179 PMC9805884

[21] Fronberg KM Bai S Teti DM. Household chaos mediates the link between family resources and child sleep. Sleep Health. (2022) 8:121–9. doi: 10.1016/j.sleh.2021.10.005 34930713 PMC8821371

[22] Whitesell CJ Crosby B Anders TF Teti DM. Household chaos and family sleep during infants' first year. J Fam Psychol. (2018) 32:622–31. doi: 10.1037/fam0000422 29781634 PMC6072580

[23] Pereira R Costa A Warkentin S Vilela S Oliveira A. Sleep duration is associated with appetitive traits in school-age years – results from the Generation XXI birth cohort. Appetite. (2024) 199:107384. doi: 10.1016/j.appet.2024.107384 38688409

[24] Zhang Z Carlson D Gilbert-Diamond D Emond JA. Associations between sleep and appetitive traits in higher-income preschoolers: a six-month study. Appetite. (2024) 196:107254. doi: 10.1016/j.appet.2024.107254 38346496 PMC11624520

[25] Matheny AP Wachs TD Ludwig JL Phillips K. Bringing order out of chaos: psychometric characteristics of the confusion, hubbub, and order scale. J Appl Dev Psychol. (1995) 16:429–44. doi: 10.1016/0193-3973(95)90028-4

[26] Wardle J Guthrie CA Sanderson S Rapoport L. Development of the children's eating behaviour questionnaire. J Child Psychol Psychiatry. (2001) 42:963–70. doi: 10.1111/1469-7610.00792 11693591

[27] Emond JA Lansigan RK Ramanujam A Gilbert-Diamond D. Randomized exposure to food advertisements and eating in the absence of hunger among preschoolers. Pediatrics. (2016) 138:e20162361. doi: 10.1542/peds.2016-2361 27940713 PMC5127075

[28] Yeum D Renier TJ Masterson TD Carlson DD Ballarino GA Lansigan RK . Genetic associations with consumption of palatable foods in the absence of hunger in response to food cues in children. Pediatr Obes. (2024) 20:e13168. doi: 10.1111/ijpo.13168 39197865 PMC11868456

[29] Keller KL Assur SA Torres M Lofink HE Thornton JC Faith MS . Potential of an analog scaling device for measuring fullness in children: Development and preliminary testing. Appetite. (2006) 47:233–43. doi: 10.1016/j.appet.2006.04.004 16828929

[30] Robinson CC Mandleco B Olsen SF Hart CH. The parenting styles and dimensions questionnaire (PSDQ). In:Perlmutter BF Touliatos J Holden GW, editors. Handbook of family measurement techniques: Volume 3. Instruments & index. Thousand Oaks, CA: Sage Publications (2001). p. 319–21.

[31] Jackson C Henriksen L Foshee VA. The authoritative parenting index: predicting health risk behaviors among children and adolescents. Health Educ Behav Off Publ Soc Public Health Educ. (1998) 25:319–37. doi: 10.1177/109019819802500307 9615242

[32] Sitnick SL Goodlin-Jones BL Anders TF. The use of actigraphy to study sleep disorders in preschoolers: some concerns about detection of nighttime awakenings. Sleep. (2008) 31:395–401. doi: 10.1093/sleep/31.3.395 18363316 PMC2276752

[33] Sadeh A Lavie P Scher A Tirosh E Epstein R. Actigraphic home-monitoring sleep-disturbed and control infants and young children: a new method for pediatric assessment of sleep-wake patterns. Pediatrics. (1991) 87:494–9. doi: 10.1542/peds.87.4.494 2011426

[34] Sadeh A Acebo C Seifer R Aytur S Carskadon MA. Activity-based assessment of sleep-wake patterns during the 1st year of life. Infant Behav Dev. (1995) 18:329–37. doi: 10.1016/0163-6383(95)90021-7

[35] ActiGraph. ActiLife 6 User's Manual [Internet]. Pensacola, FL: ActiGraph (2012). Available online at: https://s3.amazonaws.com/actigraphcorp.com/wp-content/uploads/2018/02/22094137/SFT12DOC13-ActiLife-6-Users-Manual-Rev-A-110315.pdf (Cited November 1, 2022).

[36] Cole RJ Kripke DF Gruen W Mullaney DJ Gillin JC. Automatic sleep/wake identification from wrist activity. Sleep. (1992) 15:461–9. doi: 10.1093/sleep/15.5.461 1455130

[37] Tudor-Locke C Barreira TV Schuna JM Mire EF Katzmarzyk PT. Fully automated waist-worn accelerometer algorithm for detecting children's sleep-period time separate from 24-h physical activity or sedentary behaviors. Appl Physiol Nutr Metab. (2014) 39:53–7. doi: 10.1139/apnm-2013-0173 24383507

[38] Troiano RP Berrigan D Dodd KW Mâsse LC Tilert T McDowell M. Physical activity in the United States measured by accelerometer. Med Sci Sports Exerc. (2008) 40:181–8. doi: 10.1249/mss.0b013e31815a51b3 18091006

[39] Kuczmarski RJ Ogden CL Guo SS Grummer-Strawn LM Flegal KM Mei Z . 2000 CDC Growth Charts for the United States: methods and development. Vital Health Stat. (2002) 11:1–190. 12043359

[40] Keller KL Kling SMR Fuchs B Pearce AL Reigh NA Masterson T . A Biopsychosocial model of sex differences in children's eating behaviors. Nutrients. (2019) 11:682. doi: 10.3390/nu11030682 30909426 PMC6470823

[41] Jansen E Thapaliya G Beauchemin J D'Sa V Deoni S Carnell S. The development of appetite: tracking and age-related differences in appetitive traits in childhood. Nutrients. (2023) 15:1377. doi: 10.3390/nu15061377 36986108 PMC10056659

[42] Garrett-Peters PT Mokrova I Vernon-Feagans L Willoughby M Pan Y. The role of household chaos in understanding relations between early poverty and children's academic achievement. Early Child Res Q. (2016) 37:16–25. doi: 10.1016/j.ecresq.2016.02.004 27330247 PMC4909052

[43] Zhang Z Carlson D Gilbert-Diamond D Emond JA. *The Association between Parenting Styles and Added Sugar Intake in Preschool-Aged Children* [SSRN Scholarly Paper] [Internet]. Rochester, NY: Social Science Research Network (2025). Available online at: https://papers.ssrn.com/abstract=5464118 (Cited 2026 Jan 30).

[44] Dumas JE Nissley J Nordstrom A Smith EP Prinz RJ Levine DW. Home chaos: sociodemographic, parenting, interactional, and child correlates. J Clin Child Adolesc Psychol. (2005) 34:93–104. doi: 10.1207/s15374424jccp3401_9 15677284

[45] Koo TK Li MY. A guideline of selecting and reporting intraclass correlation coefficients for reliability research. J Chiropr Med. (2016) 15:155–63. doi: 10.1016/j.jcm.2016.02.012 27330520 PMC4913118

[46] Evans GW. Child development and the physical environment. Annu Rev Psychol. (2006) 57:423–51. doi: 10.1146/annurev.psych.57.102904.190057 16318602

[47] Barnes J Gardiner J Sutcliffe A Melhuish E. The parenting of preschool children by older mothers in the United Kingdom. Eur J Dev Psychol. (2014) 11:397–419. doi: 10.1080/17405629.2013.863728

[48] Blair C. Stress and the development of self-regulation in context. Child Dev Perspect. (2010) 4:181–8. doi: 10.1111/j.1750-8606.2010.00145.x 21779305 PMC3138186

[49] Hong Y McCormick SA Deater-Deckard K Calkins SD Bell MA. Household chaos, parental responses to emotion, and child emotion regulation in middle childhood. Soc Dev Oxf Engl. (2020) 30:786. doi: 10.1111/sode.12500 34334970 PMC8323614

[50] Pink AE Lee M Price M Williams C. A serial mediation model of the relationship between alexithymia and BMI: the role of negative affect, negative urgency and emotional eating. Appetite. (2019) 133:270–8. doi: 10.1016/j.appet.2018.11.014 30465802

[51] von Stumm S. Adolescents' perceptions of household chaos predict their adult mental health: a twin-difference longitudinal cohort study. Psychol Sci. (2024) 35:736–48. doi: 10.1177/09567976241242105 38717488 PMC13020938

[52] Goossens L Van Malderen E Van Durme K Braet C. Loss of control eating in adolescents: associations with adaptive and maladaptive emotion regulation strategies. Eat Behav. (2016) 22:156–63. doi: 10.1016/j.eatbeh.2016.06.015 27289522

[53] Zhang X. Household chaos and caregivers' and young children's mental health during the COVID-19 pandemic: a mediation model. J Child Fam Stud. (2022) 31:1547. doi: 10.1007/s10826-022-02283-4 35287306 PMC8907899

[54] Evans GW. The physical context of child development. Curr Dir Psychol Sci. (2021) 30:41–8. doi: 10.1177/0963721420980719

[55] Kawai M. Disruption of the circadian rhythms and its relationship with pediatric obesity. Pediatr Int. (2022) 64:e14992. doi: 10.1111/ped.14992 34525248

[56] Adnan D Trinh J Bishehsari F. Inconsistent eating time is associated with obesity: a prospective study. EXCLI J. (2022) 21:300. doi: 10.17179/excli2021-4324 35368461 PMC8971321

[57] Hohman E Hernandez E Dadzie A Master L Anzman-Frasca S Paul I . 0101 Greater variability in sleep health is associated with obesogenic appetitive traits in 6-year-olds in the INSIGHT study. Sleep. (2023) 46:A45–6. doi: 10.1093/sleep/zsad077.0101

[58] Podsakoff PM Podsakoff NP Williams LJ Huang C Yang J. Common method bias: it's bad, it's complex, it's widespread, and it's not easy to fix. Annu Rev Organ Psychol Organ Behav. (2024) 11:17–61. doi: 10.1146/annurev-orgpsych-110721-040030

