# Supplementary material for: Associations between household chaos and appetitive traits in preschoolers and preadolescents
Source: Front Public Health. 2026 Sep 21;14:1800392. doi: 10.3389/fpubh.2026.1800392 (PMC13635114; doi:10.3389/fpubh.2026.1800392)
Supplement: Supplementary file 1 [file Data_Sheet_1.pdf]

Supplementary Material

Associations Between Household Chaos and Appetitive Traits in Preschoolers and Preadolescents

Authors: Zhuoya Zhang[1*], Delaina Carlson[1,2], Caroline Borowy[1], Mingliang Ge[1], Diane Gilbert-Diamond[1,3,4], Jennifer A. Emond[3,5]

Affiliations:
[1]Department of Epidemiology, Geisel School of Medicine, Dartmouth College, NH 03755, USA
[2]Department of Human Development and Family Sciences, University of Connecticut, CT 06269, USA
[3]Department of Pediatrics, Geisel School of Medicine, Dartmouth College, NH 03756, USA
[4]Department of Medicine, Geisel School of Medicine, Dartmouth College, NH, 03756, USA
[5]Department of Biomedical Data Science, Geisel School of Medicine, Dartmouth College, NH, 03756, USA

Correspondence
Zhuoya Zhang
zhuoya.zhang.gr@dartmouth.edu

Keywords: household chaos, appetitive trait, childhood obesity, preschool, adolescence, eating behavior.

Figure S1. Hypothesized relationship between household chaos (exposure variable), child appetitive traits (outcome variables), and covariates (child, parent, and household characteristics).

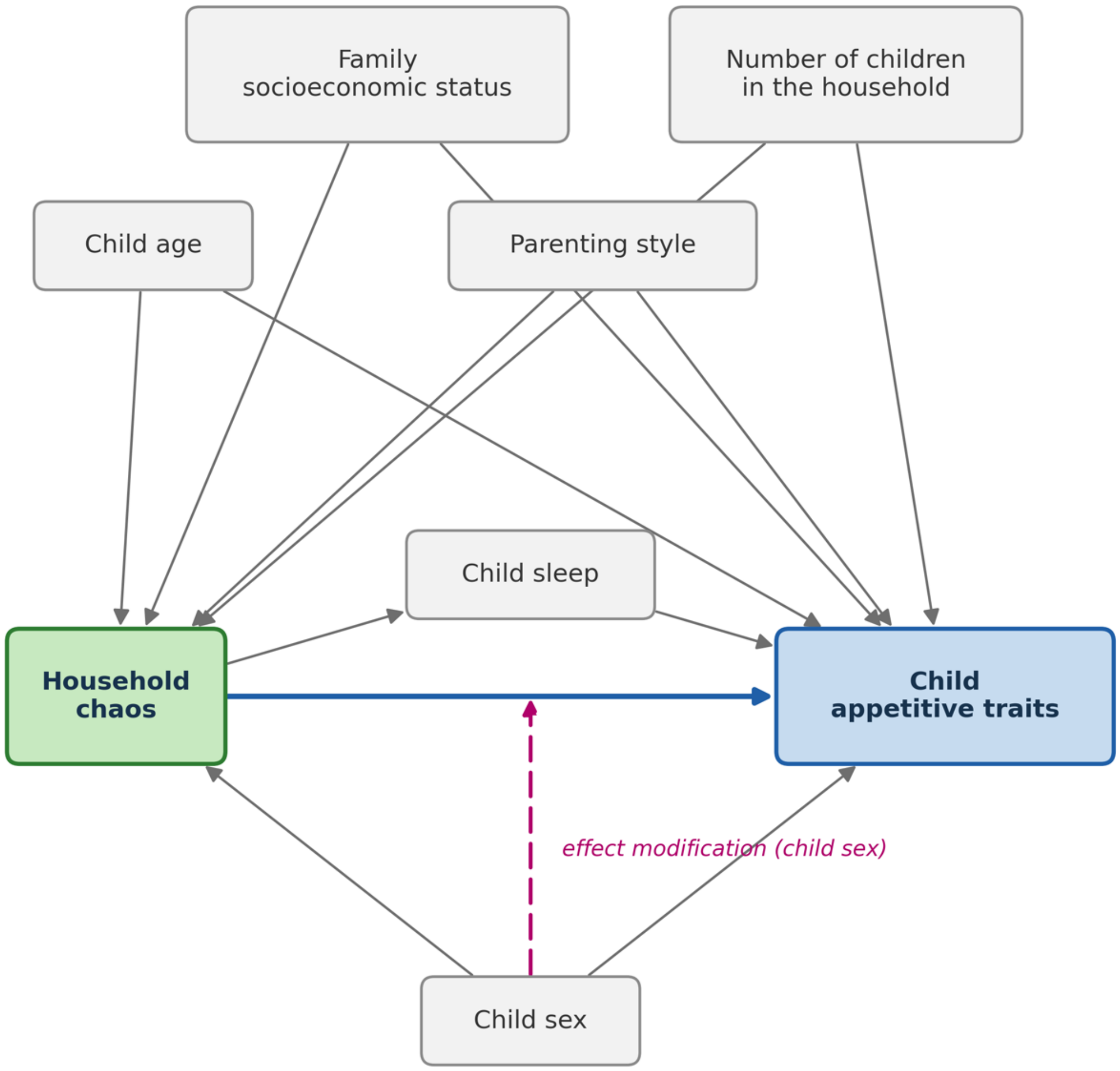

Figure S2. Bivariate analysis between household chaos, child sleep, appetitive traits, and weight status at baseline in Study A preschoolers (N=92). *Note*: Pearson's correlation coefficients (*r)* are presented. * *P*-value < 0.05 from Pearson's correlational test.

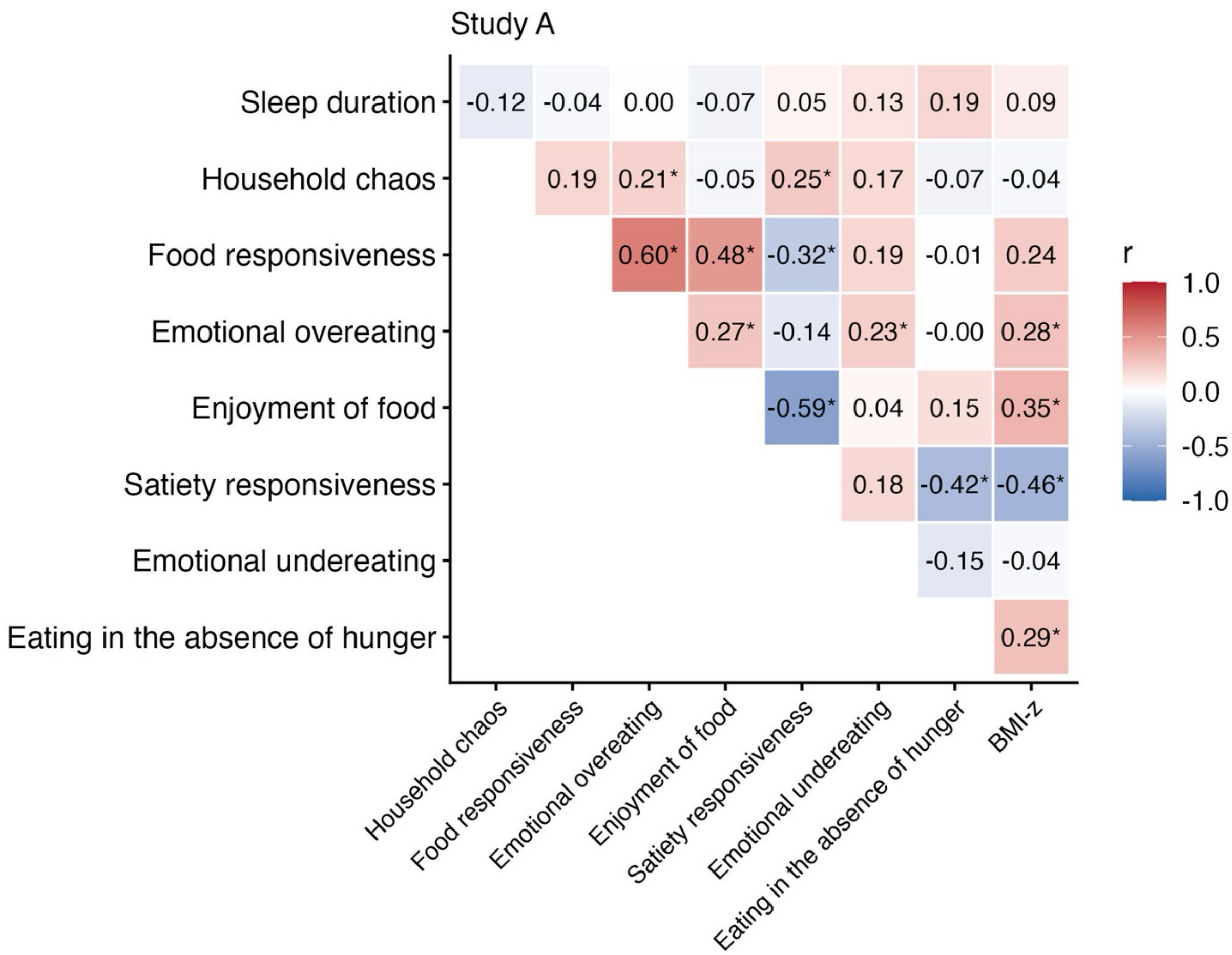

Figure S3. Bivariate analysis between household chaos, child sleep, appetitive traits, and weight status at baseline in Study B pre-adolescents (N=184). *Note*: Pearson's correlation coefficients (*r*) are presented. * *P*-value < 0.05 from Pearson's correlational test.

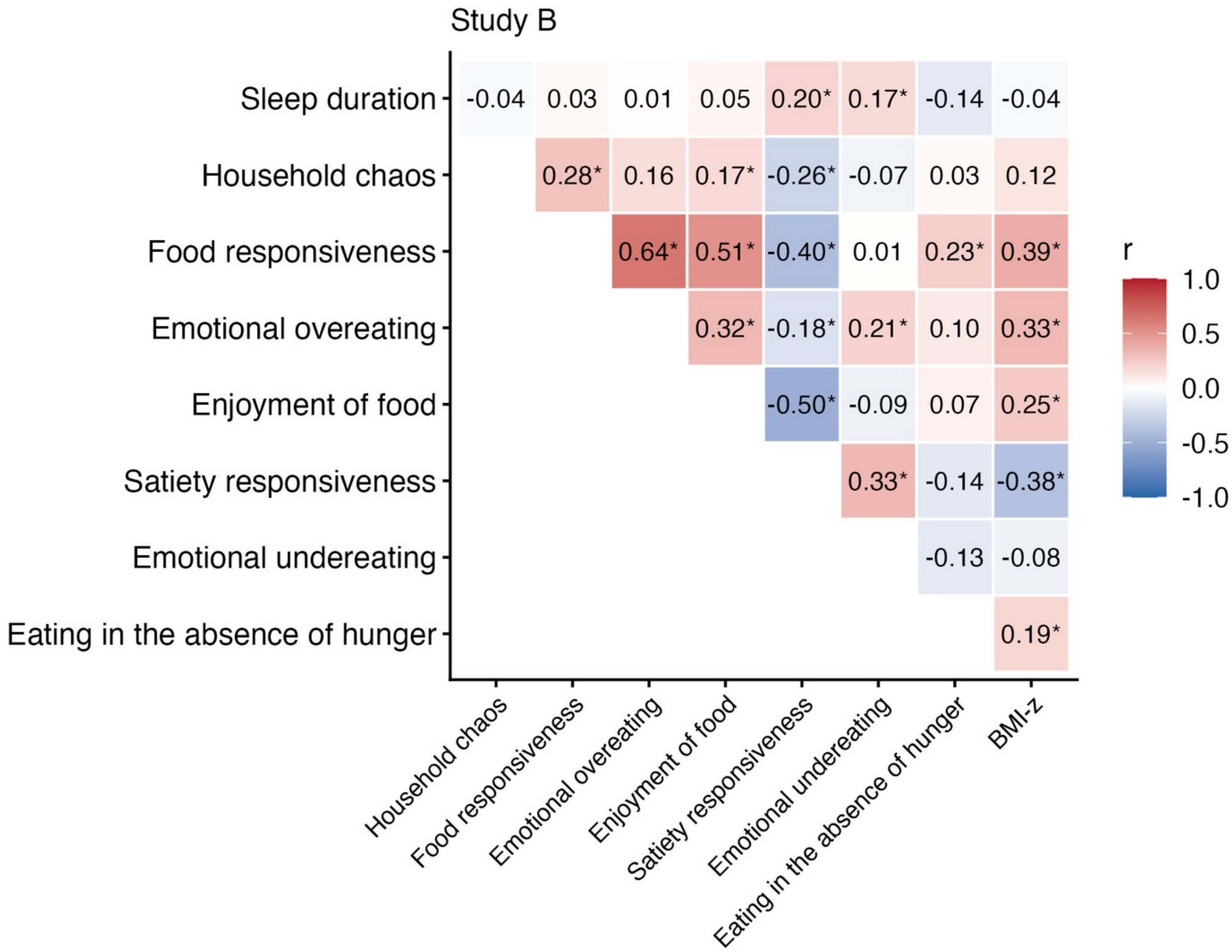

Table S1. Participant baseline characteristics between the baseline and follow-up samples, presented as mean ± SD or N (%).

	**Study A: Preschoolers**			**Study B: Preadolescents**		
	Baseline participants	*Six-month follow-up participants*		*Baseline participants*	*One-year follow-up participants*	
	N = 92	N = 80	p^c	N = 184	N = 95	p^c
Child age (years)	4.2 ± 1.0	4.2 ± 1.0	0.89	10.9 ± 1.2	10.9 ± 1.2	0.99
Female	40 (44%)	37 (46%)	0.83	77 (42%)	36 (38%)	0.74
White non-Hispanic	81 (88%)	70 (88%)	0.99	166 (90%)	90 (95%)	0.28
Household income ($)[a]			0.97			0.76
<65000	26 (28%)	20 (25%)		24 (13%)	9 (10%)	
65000-145000	47 (51%)	43 (54%)		99 (54%)	51 (54%)	
145000-225000	13 (14%)	12 (15%)		43 (23%)	23 (24%)	
>225000	6 (7%)	5 (6%)		18 (10%)	12 (13%)	
Number of children in the household[b]			0.99			-
1	16 (17.4%)	14 (17.5%)		-	-	
2	46 (50.0%)	39 (48.8%)		-	-	
3	15 (16.3%)	13 (16.3%)		-	-	
4 or more	15 (16.3%)	14 (17.5%)		-	-	

Notes:
[a]Household income was missing for 4 participants in Study A and was imputed with the sample mode.
[b]The number of children in the household was only collected in Study A.
[c]*P*-values are computed with Welch's two-sample t-test or Pearson's Chi-squared tests.

Table S2. Internal consistency of the questionnaires used in the study.

**Instrument**	**Subscale**	**Number of Items**	**Study A: Preschoolers (N = 92)**	**Study B: Preadolescents (N = 184)**
			Cronbach's α	Cronbach's α
Child Eating Behavior Questionnaire	Food responsiveness	5	0.77	0.83
	Emotional overeating	4	0.80	0.82
	Enjoyment of food	4	0.91	0.82
	Satiety responsiveness	5	0.82	0.80
	Emotional undereating	4	0.69	0.77
Confusion, Hubbub, and Order Scale	Overall	15	0.85	0.87
Parenting Style and Dimensions Questionnaire	Authoritative parenting	15	0.85	NA
	Permissive parenting	5	0.68	NA
Authoritative Parenting Index	Demandingness	4	NA	0.52
	Responsiveness	5	NA	0.75

Table S3. Distribution of household chaos by participant baseline demographics in preschoolers (Study A, N = 92).

		CHAOS score[a]		
	**N**	**Mean**	**SD**	***p***[b]
**Child age**				
≥3 and <4	28	28.71	6.90	0.72
≥4 and <5	27	29.78	6.27	
≥5 and <6	28	25.71	6.56	
≥6 and <7	9	29.78	5.49	
**Child sex**				
Female	40	27.93	5.65	0.95
Male	52	28.44	7.32	
**Child race/ethnicity**				
White, non-Hispanic	81	28.16	6.43	0.95
Other	11	28.64	8.19	
**Annual household income ($)**				
<65,000	26	29.15	7.75	0.45
65,000-145,000	47	28.15	6.26	
145,000-225,000	13	27.69	6.94	
>225,000	6	25.83	2.64	
**Number of children living in the household**				
1	16	22.50	3.35	<0.0001
2	46	27.61	5.19	
3	15	30.53	6.22	
4 or more	15	33.87	8.27	

Notes:
[a]CHAOS, Confusion, Hubbub, and Order Scale (range: 15-60).
[b]*P*-values were computed via the Spearman correlation test for continuous variables (i.e., child age), the Wilcoxon rank sum test with continuity correction for categorical variables with two levels (i.e., child sex and race/ethnicity), or the Jonckheere-Terpstra test for trend for ordinal variables with more than two levels (i.e., annual income and number of children in the household).

Table S4. Distribution of household chaos by participant baseline demographics in preadolescents (Study B, N = 184).

		CHAOS score[a]		
	**N**	**Mean**	**SD**	***p*[b]**
**Child age**				
≥ 8 and <9	2	27.00	4.24	0.81
≥ 9 and <10	55	27.71	5.80	
≥ 10 and <11	34	29.38	6.46	
≥ 11 and <12	57	28.40	8.24	
≥ 12 and <13	36	28.97	7.73	
**Child sex**				
Female	77	28.22	7.65	0.58
Male	106	28.71	6.69	
NA	1	23	NA	
**Child race/ethnicity**				0.37
White, non-Hispanic	166	28.27	7.02	
Other	18	30.33	7.57	
**Annual household income ($)**				
<65,000	24	30.29	8.31	0.003
65,000-145,000	99	29.10	6.47	
145,000-225,000	43	27.16	7.67	
> 225,000	18	25.72	6.38	

Notes:
[a]CHAOS, Confusion, Hubbub, and Order Scale (range: 15-60).
[b]*P*-values were computed via the Spearman correlation test for continuous variables (i.e., child age), the Wilcoxon rank sum test with continuity correction for categorical variables with two levels (i.e., child race/ethnicity), the Kruskal-Wallis rank sum test for categorical variables with more than two levels (i.e., child sex), or the Jonckheere-Terpstra test for trend for ordinal variables with more than two levels (i.e., household income).

Table S5. Summary statistics and stability of household chaos, child appetitive traits, and BMI-z across time.

Variable	Study A: Preschoolers					Study B: Preadolescents				
	Baseline		Six-Month Visit			Baseline		One-Year Visit		
	N	Mean ± SD	N	Mean ± SD	ICC[e]	N	Mean ± SD	N	Mean ± SD	ICC[e]
CHAOS[a]	92	28.2 ± 6.61	80	27.8 ± 6.59	0.93	184	28.5 ± 7.08	95	28.4 ± 7.45	0.85
Food responsiveness[b]	92	2.35 ± 0.69	80	2.38 ± 0.72	0.77	184	2.58 ± 0.74	95	2.55 ± 0.80	0.74
Emotional overeating	92	1.56 ± 0.53	80	1.68 ± 0.57	0.61	184	2.21 ± 0.66	95	2.31 ± 0.75	0.59
Enjoyment of food	92	3.70 ± 0.80	80	3.62 ± 0.68	0.76	184	3.94 ± 0.59	95	3.89 ± 0.58	0.65
Satiety responsiveness	92	3.22 ± 0.71	80	3.19 ± 0.58	0.68	184	2.76 ± 0.64	95	2.72 ± 0.60	0.71
Emotional undereating	92	2.54 ± 0.72	80	2.40 ± 0.88	0.56	184	2.93 ± 0.72	95	2.89 ± 0.68	0.59
EAH (calories)[c]	52	79.17±71.47	37	86.29 ± 53.17	0.63	184	440.00 ± 222.06	94	509.16 ± 238.32	0.51
BMI-z[d]	52	0.40 ± 1.06	NA	NA	NA	183	0.48 ± 1.00	94	0.48 ± 1.01	0.94

Notes:
[a]CHAOS, Confusion, Hubbub, and Order Scale (range: 15-60).
[b]Appetitive traits (parent reported) were assessed via the Child Eating Behavior Questionnaire subscales (range: 1 to 5).
[c]EAH, eating in the absence of hunger was operationalized as the caloric intake during the EAH experiment.
[d]BMI-z, age- and sex-standardized body mass index z-score based on the CDC 2000 growth chart.
[e]ICC, intra-class correlation (two-way random-effect model).

Table S6. Sensitivity analysis: associations between household chaos, child appetitive traits, and BMI-z, further adjusting for family structure, nighttime sleep duration, and parenting style in preschoolers (Study A).

	Main model[a]			Main model + number of children living in the household			Main model + nighttime sleep duration			Main model + parenting style		
**Outcome variable**	N	β_s[b]	95% CI	N	β_s	95% CI	N	β_s	95% CI	N	β_s	95% CI
Food responsiveness	92	0.10	-0.07, 0.27	92	0.08	-0.11, 0.27	81	0.09	-0.00, 0.39	92	0.08	-0.09, 0.26
Emotional overeating	92	0.18	0.00, 0.35[c]	92	0.14	-0.06, 0.34	81	0.18	-0.02, 0.38	92	0.15	-0.03, 0.33
Enjoyment of food	92	-0.02	-0.20, 0.15	92	-0.01	-0.20, 0.18	81	0.10	-0.11, 0.30	92	-0.01	-0.18, 0.17
Satiety responsiveness	92	0.20	0.03, 0.37	92	0.24	0.05, 0.43	81	0.13	-0.08, 0.33	92	0.18	0.01, 0.35
Emotional undereating	92	0.08	-0.09, 0.25	92	0.10	-0.10, 0.29	81	0.11	-0.08, 0.31	92	0.06	-0.12, 0.23
Eating in the absence of hunger	70	-0.03	-0.25, 0.18	70	-0.04	-0.30, 0.22	65	0.04	-0.19, 0.26	70	-0.06	-0.28, 0.16
BMI-z[d]	52	-0.02	-0.31, 0.27	52	0.02	-0.37, 0.42	44	0.01	-0.32, 0.34	52	-0.02	-0.32, 0.27

Notes:
[a]The main models were adjusted for child age, sex, and annual household income.
[b]Standardized beta coefficients and 95% confidence intervals are presented in the table.
[c]$p = 0.044$.
[d]BMI-z: age- and sex-adjusted body mass index z-scores.

Table S7. Sensitivity analysis: associations between household chaos, child appetitive traits, and BMI-z, further adjusting for parenting style and nighttime sleep duration in preadolescents (Study B).

**Outcome variable**	**Main model**[a]			**Main model + parenting style (responsive and demanding parenting)**			**Main model + responsive parenting**			**Main model + nighttime sleep duration**		
	**N**	β_s[b]	**95% CI**	**N**	β_s[b]	**95% CI**	**N**	β_s[b]	**95% CI**	**N**	β_s[b]	**95% CI**
Food responsiveness	184	0.21	0.09, 0.32	184	0.20	0.08, 0.32	184	0.20	0.09, 0.32	157	0.22	0.10, 0.35
Emotional overeating	184	0.19	0.07, 0.31	184	0.18	0.05, 0.30	184	0.18	0.05, 0.30	157	0.19	0.05, 0.32
Enjoyment of food	184	0.07	−0.06, 0.20	184	0.08	−0.05, 0.21	184	0.08	−0.05, 0.21	157	0.13	−0.01, 0.26
Satiety responsiveness	184	−0.14	−0.25, −0.02	184	−0.16	−0.28, −0.04	184	−0.15	−0.27, −0.03	157	−0.16	−0.29, −0.04
Emotional undereating	184	0.00	−0.12, 0.13	184	−0.02	−0.15, 0.11	184	−0.01	−0.14, 0.12	157	−0.01	−0.15, 0.13
Eating in the absence of hunger	184	0.06	−0.06, 0.18	184	0.07	−0.05, 0.19	184	0.07	−0.06, 0.19	157	0.03	−0.11, 0.16
BMI-z[c]	183	0.03	−0.05, 0.12	183	0.04	−0.05, 0.12	183	0.03	−0.05, 0.11	156	0.06	−0.03, 0.15

Notes:

[a] Main models were adjusted for child age, sex, and annual household income.

[b] Standardized beta coefficients (β_s) and 95% confidence intervals are presented in the table.

[c] BMI-z: age- and sex-adjusted body mass index z-scores.

Table S8. Associations between household chaos, child appetitive traits, and BMI-z.

	Study A: Preschoolers				Study B: Preadolescents			
**Outcome variable**	N	Number of observations	β_s[a]	95% CI	N	Number of observations	β_s	95% CI
Food responsiveness	92	172	0.10	-0.07, 0.27	184	279	0.21	0.09, 0.32
Emotional overeating	92	172	0.18	0.00, 0.35[b]	184	279	0.19	0.07, 0.31
Enjoyment of food	92	172	-0.02	-0.20, 0.15	184	279	0.07	-0.06, 0.20
Satiety responsiveness	92	172	0.20	0.03, 0.37	184	279	-0.14	-0.25, -0.02
Emotional undereating	92	172	0.08	-0.09, 0.25	184	279	0.00	-0.12, 0.13
Eating in the absence of hunger	70	89	-0.03	-0.25, 0.18	184	278	0.06	-0.06, 0.18
BMI-z[c]	52	52	-0.02	-0.31, 0.27	183	277	0.03	-0.05, 0.12

Notes:
[a]β_s: Standardized beta coefficients and 95% confidence intervals are presented in the table. Models were adjusted for child age, sex, and annual household income.
[b]$p = 0.044$.
[c]BMI-z: age- and sex-adjusted body mass index z-scores. Study A collected anthropometry only in 52 participants at baseline in-person visits, because in-person visits were paused during the COVID-19 pandemic.

Table S9. Effect modification by child sex (household chaos × sex interaction).

	**Study A: Preschoolers (N=92)**		**Study B: Preadolescents (N=184)**	
**Appetitive trait**	**β_s (95% CI)**	***p***	**β_s (95% CI)**	***p***
Food responsiveness	−0.23 (−0.58, 0.12)	0.20	0.14 (−0.09, 0.38)	0.23
Emotional overeating	−0.09 (−0.45, 0.27)	0.64	0.08 (−0.17, 0.33)	0.53
Enjoyment of food	−0.03 (−0.39, 0.32)	0.86	−0.18 (−0.44, 0.07)	0.17
Satiety responsiveness	0.02 (−0.33, 0.38)	0.90	0.16 (−0.08, 0.40)	0.19
Emotional undereating	−0.10 (−0.45, 0.25)	0.58	0.16 (−0.10, 0.42)	0.22
Eating in the absence of hunger	−0.13 (−0.55, 0.30)	0.56	0.04 (−0.21, 0.28)	0.78

Notes: Values are standardized interaction coefficients (β_s) for household chaos × child sex (reference = male), with 95% confidence intervals and p-values.
